# The design and equipments of hospital pharmacies in Isfahan, Iran

**Published:** 2010

**Authors:** Ali Mohammad Sabzghabaee, Shirinsadat Badri, Hossein Esna Ashari, Seyyed Mahmood Hosseini

**Affiliations:** a Clinical Toxicology Research Center, Isfahan University of Medical Sciences, Isfahan, Iran; b Resident of Clinical Pharmacy, Department of Pharmacotherapy, Tehran University of Medical Sciences, Tehran, Iran; c Department of Research and Development, Vice-Chancellery for Administration and Financial Affairs, Isfahan University of Medical Sciences, Isfahan, Iran; d Pharmacy Student, School of Pharmacy and Pharmaceutical Sciences, Isfahan University of Medical Sciences Isfahan, Iran

**Keywords:** Hospital Pharmacy, Pharmacy Management, Pharmacy Design, Isfahan

## Abstract

**BACKGROUND:**

Nowadays pharmaceutical care departments located in hospitals are amongst the important pillars of the healthcare system. The aim of this study was to evaluate designing features and equipments of hospital drugstores affiliated with Isfahan University of Medical Sciences.

**METHODS:**

In this cross-sectional study a self-defined and validated questionnaire was used which included all the necessary and standard needed spaces and equipments of an ideal hospital pharmacy. The questionnaire was filled in by one of the researchers in all twelve hospital drugstores located in the teaching and non-teaching hospitals affiliated with Isfahan University of Medical Sciences. Data analysis was done using SPSS (version 14).

**RESULTS:**

Results showed that 56% of drugstore space allocations were unsuitable. Used pharmaceutical equipments in 75% of surveyed hospitals were not according to the standards. Almost all of these pharmacies had rather an enough space for storage, but cold storages were not designed in 58% of them. In 66% of perused hospitals, pharmaceutical services disposal level was admissible. The structural engineering parameters like size and dimensions, available spaces, availability of structural planes, existence of air conditioning systems and brightness controllers, adequate stores for drugs and safe places for narcotics were observed in 55% of pharmacies.

**CONCLUSIONS:**

There are apparent out of standard space allocations and shortages of needed equipments for offering drug services in studied drugstores that may probably lead to a waste of time and money. These issues may reduce the efficiency and safety of pharmaceutical services and drug administration in hospitals.

Surprisingly, there are still some physicians in Iran who think about a big store of drugs, when they hear the words “hospital pharmacy”. Their day to day hospital care experiences had shown them before, that the only thing which is expected from a hospital pharmacy is supplying medicines. Many of the hospital mangers in Iran who are basically supposed to be physicians do think the same.^1^ In many developing countries today, modern and advanced healthcare system is benefited from hospital drugstores and clinical pharmacists as great invaluable elements in the curing process.^2^,^3^ Branches of a standard hospital pharmacy are recommended to be wide spread in different departments of the hospital for ensuring the high quality pharmaceutical services.^4^ Proficient design of needed rooms and spaces, plays a basic role to reach standard scientific pharmaceutical care services.^5^ There are particular standards for hospital pharmacy administration in Iran in addition to some efficient and interesting worldwide ones. These standards can be used after being modified according to our cultural and even religious desires.^6^

A survey done by Malik et al showed that the most important problems in hospital pharmacies management are dearth of personnel, equipments and spaces deficiency and warranty of persuading continuous drug services.^7^

There are few published studies done in Iran on this case. In a study done by Vaziri on selected hospitals affiliated with the Iranian Social Security Organization (ISSO), lots of deficiencies and inelegances was subjectively observed in equipments, desired spaces and dispersing processes that definitely cause a waste in human resources and the organization funds, and may consequently impose a reduction in ISSO services proficiency and safety too.^8^ In another study Mortazavi et al emphasized on a huge gap between hospital pharmacy services represented in 12 surveyed hospitals affiliated with Shahid Beheshti University of Medical Sciences and the standard guidelines.^1^ Inadequacy of the needed space for professional services, non-standard equipments and instruments and low rate of (clinical) pharmacists effective attendance in clinical wards were reported to be remarkable problems for establishing a standard scientific pharmaceutical care service.^1^

On the way of moving from traditional hospital pharmacies to the more scientific and modern ones, it seems crucial to have a situation analysis before making any decision on this important issue. Isfahan is the second most populated city in Iran and is considered a well-developed province around the country as well. Despite the essential role of administrative authorities of this province on the health care system of the country, to our best of knowledge, there is no previously published research on the basic needs of the above mentioned movement. The aim of this study was to evaluate the physical design and available equipments of the in-patient pharmacies located in hospitals affiliated with Isfahan University of medical sciences (IUMS). The words “hospital pharmacy” and “drugstore” are used interchangeably within this article and “pharmaceutical care department” is the ideal scientific gold standard terminology for them.

## Methods

This study was done on the all 12 teaching and non-teaching hospitals affiliated with IUMS in 2007-2008. Both categories (of teaching and non-teaching hospitals) were included in the study because all of the in-patient hospital pharmacies in both types were not teaching ones (for pharmacy students). The full names of hospitals are not mentioned here for the sake of privacy but they were two psychiatric hospitals (PSY1 and PSY2), a women general teaching hospital (WMN), a cardiology specialized teaching hospital (CRD), an oncologyhematology specialized hospital (ONH), an ophthalmology specialized teaching hospital (OPH), a burn and trauma specialized teaching hospital (BRT), an orthopedic specialized hospital (ORT), a general teaching medical center (GMC) and finally three other general hospitals (GNH1, GNH2, and GNH3). The design of study was cross-sectional and the descriptive data was collected using a questionnaire which was validated (face and content validity) using focus group method^9^ filled in on the basis of direct observation and peruse by one of the authors in the field. The questionnaire was divided into 7 separated fields: 1) general questions about the surveyed hospitals, 2) questions evaluating the hospital pharmacy including questions about the level of offered services, number of employee’s, total pharmacy surface area, etc 3) questions about the accessibility of the pharmacy by health care professionals and patients 4) questions about the expected spaces in a standard desirable drugstore, 5) questions about needed equipments in a standard desirable pharmacy, 6) questions about suitable physical structure for a standard drugstore, and 7) questions about drug storing system. The total number of questions in this questionnaire was 50. According to available standard references,^5^,^10^,^11^ scientifically accepted space allocation for a standard general hospital pharmacy was summarized (Table 1) and relevant subjects of the evaluated hospital pharmacies were compared to it, whether it was more than enough, acceptable or unacceptable. Descriptive analysis of the data was done using SPSS (version 14).

**Table 1:** Required hospital pharmacy spaces for offering technical services *

Number of patients bed	Surface area needed (m^2^)		
	50 beds	100 beds	200 beds
Dispensing area	25	32	50
IV Drug admixture area	-	18	20
Stores	-	12	20
Compounding area	-	-	12
Logistic area	-	-	10
Circulation area	-	-	6

* Adopted from references 5, 11

## Results

According to the present findings, 25% of surveyed drugstores had a 24-hour service. This could cause problems with preparing and surveillance of needed drugs or drug counseling by the pharmacist. Also, the number of pharmacists in each one of the studied pharmacies-except GMT hospital drugstore with 3 staff pharmacists- was not more than 2. On the other hand, the ratio between the number of pharmacists and the number of hospital beds was not in concordance with the accepted pharmaceutical care standards.^6^ Assessment of joint drugstores, offering service in both outpatient (clinic and emergency wards) and inpatient departments showed a percentage of 92. In 56% of observed hospitals the operating spaces were standard.

There were enough store space (according to table 1 which mentions the standards) in almost all pharmacies, but 58% of them had no cool or cold stores. The rest room and counseling office were not planned in PSY1, PSY2, GNH1, GNH2, ORT and WMN hospital pharmacies. Used equipments in more than 75% of studied hospitals were not adequate and were less than standard; 50% of drugstores had a thermometer built-in refrigerator; 66% had no sanitary sink outfitted with hot and cold water, 92% had no cupboards, 100% had open cabinets, 83% had no container used to prepare syrups or compounding drugs, 100% had no autoclave, 75% had classified cabinets, 92% didn’t have resistant desk to chemicals, 75% had no balance, 92% had no distillated water maker, 100% had no capsule filler machine, 100% didn’t have a pressure pump, 100% didn’t have a vacuum pump, 92% had no laboratory heater, 100% had no condensometer, 100% didn’t have a pH-meter, 92% didn’t have laboratory tools (such as test tube, pipette, etc), 75% didn’t have fire and smoke alarming or firefighting systems (of course, one or two firefighting capsules existed in most of drugstores), 92% didn’t have a ladder or lifter for upper cabinets accessibility (a chair or a long bar is used to help employees use upper cabinets), 75% had no notice-board, 67% had archive documents, 75% didn’t have a seat for clients while waiting to take drugs and 92% didn’t have co-didactic tools (like posters, etc).

There were computer systems in all surveyed drugstores but there is no special efficacy for these instruments without internet connection or medical and drug information banks accessibility. Drug information delivery was documented in 66% of studied hospitals but some hospitals such as GMC, PSY1, PSY2, GNH1 and WMN had poor pharmaceutical services which might be due to the long physical distance between clinical wards and drugstore. This caused some access problems for hospital personnel (e.g. physicians, nurses …) and clients as well. The structural engineering parameters like size and dimensions, available spaces, availability of structural plans, existence of air conditioning systems and brightness controllers, adequate stores for drugs and safe places for opiates were observed in 55% of pharmacies. In all pharmacies the stores used the standard First In-First Out (FIFO) system.

**Figure 1:**
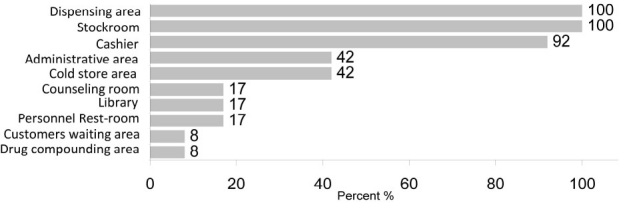
Frequency of necessary spaces in hospital pharmacies affiliated with IUMS

**Figure 2:**
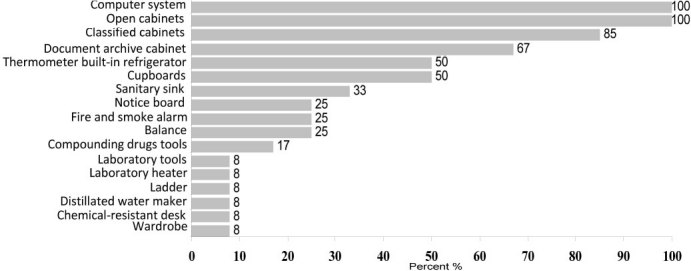
Frequency of necessary equipments in hospital pharmacies affiliated with IUMS

Although in almost all stores the drugs classified in separate cupboards, the ratio of drug mass and cupboard’s capacity was not proportional and adequate (1.9-3.6 per unit of drug). There were no firefighting or other safety equipments in the pharmacies. The results are summarized and categorized in figures 1 and 2.

## Discussion

Hospital pharmacy (pharmaceutical care department) is defined as a dynamic and scientific unit which prepares, reserves, compounds, packs and disperses drugs for both inpatients and out-patients in a hospital.^12^ Furthermore, drug control and information services are offered there. The hospital drugstore unit must be easy to access for the departments it offer services. The best place for this unit is the first-floor which is accessible for in-patient, emergency and out-patient departments. Moreover, it gives a better drug receiving availability for purchased drug products.^10^ Unfortunately the present findings showed that in the studied hospitals this important department is almost always located in an unsuitable and nearly worthless area of the hospital mostly in the underground floor or near the central stores of the institution. This may be considered to be the major cause of trouble in providing pharmaceutical care services.

Required pharmacy space depends on the number and variety of clinical departments and the number of hospital beds. The minimum area for a hospital pharmacy is 25 m^2^. There is a list of hospital drugstore needed spaces in a 50, 100 and 200 bed hospital in table 1. The required area for pharmacy is 0.4 m^2^-0.5 m^2^ per hospital bed. In larger hospitals an extra 0.5 m^2^ per hospital bed is suggested. The required store space in larger hospitals is 0.3 m^2^-0.4 m^2^ for each hospital bed that gives a 17 m^2^ area per 100 beds.^11^,^13^ These figures are so far from the allocated spaces in IUMS affiliated pharmacies which show a long way for getting near the standards.

General equipments needed in a typical hospital pharmacy include: balances, cabinets (made by wood or metal), distillated water maker, filters, compounding containers, autoclave, capsule filling machines, heater, pressure pump, vacuum pump, refrigerator, condensometer, and pH-meter.^6^ Hospital pharmacy official equipments include: library, desk, chair, notice-board, computer, printer, archive documents, cupboard, etc. As figure 2 shows, again most of our hospital pharmacies are suffering from shortage of the primary and necessary equipments. Unquestionably this fact influences the quality of pharmaceutical care which is expected from the mentioned department.

According to the present findings, there were no drugstores in which more than two pharmacists working on the same working period. Obviously, the lower number of pharmacists causes the lower level and lower quality of pharmaceutical care giving. As a comparable example there are 25 pharmacists working in an American hospital with 1100 beds and the number of employed staff pharmacists in a 350 beds Christian hospital was reported to be 12 people in 3 shifts.^14^ So, expecting standard and appropriate services in general teaching hospitals like GMC, GNH2 and even PSY2 with 950, 394 and 417 permanent beds (761, 280 and 195 active beds) while employing just 2 pharmacists looks unwise and needs serious reviews. Other important points are lack of 56% of standard physical space and lack of standard tools by 75%. This is in contrast with similar hospital pharmacies even in neighborhood countries e.g. Indian Christian hospitals in India, with 90% standard spaces and equipments.^14^ Deficiencies in surveyed hospitals’ suitable space and tools have important effects on pharmacy proficiency, leading to disability of keeping, tenet classifying and preparing adequate drugs causing personnel dissatisfaction. Existence of the manager office and a counseling room for patients, nurses or physicians is one of the most important needs which are not planned in lots of cases.

The other problem in most of the studied pharmacies was lack of rich and appropriate scientific references and resources about pharmacotherapy, drug interactions or drug information. Not offering a 24-hour drug service by 75% of hospital pharmacies is another chief problem. There was no pharmacist working in plenty of them after official time (even at evening working shift). It is necessary for a capable drug system to have pharmacists servicing and drug counseling to physicians and nursing team for all round the day and night in 3 shifts. Another problem was limited standard and necessary tools in pharmacies. For example there was no ladder or lifter to access upper cabinets. A chair or long bar was often used to help employees in using upper cabinets. Besides, tools and rooms required for preparing compounding drugs existed only in 17% of studied drugstores. All stores used FIFO standard method. Also, only 55% of drugstores had the proper structural situations and just 86% of them followed drug dispensing standard process.

## Conclusions

This study denoted lots of deficiencies and inelegances in spaces, equipments, services and drug counseling which is supposed to be offered by the pharmaceutical care departments in the studied hospitals. This may cause a waste of money and human resources, leading to limitations in offering safe and scientific pharmaceutical care services. This matter manifests great differences between our situation and accepted global standards. It looks necessary to have systematic programs to eliminate this kind of problems. It is believed that a very important recommendation for prevention and solving of these kinds of concerns is to employ enough number of clinical/hospital pharmacy specialists as the administrator or manager of the pharmacies located in hospitals. They are supposed to follow-up the professional standards of hospital pharmacy practice including the correct and scientific method of space and resource allocation. This approach has been started in Iran from 1995 by training the needed specialized personnel (clinical pharmacists) and entrusting the whole management of hospital pharmacies to them. Obviously, a fully word by word translated curriculum of clinical/hospital pharmacy training courses is not expected to fulfill the local need and will not help to eradicate the above mentioned problems in hospital pharmacy management. The same story may be expected for the hospital pharmacies located in other middle-east countries.
